# Analgesic effect of S (+)‐flurbiprofen plaster in a rat model of knee arthritis: analysis of gait and synovial fluid prostaglandin E_2_ levels

**DOI:** 10.1111/jphp.12914

**Published:** 2018-04-01

**Authors:** Ayaka Fukumoto, Kyoko Tajima, Miyuki Hori, Yoshihisa Toda, Shinsuke Kaku, Hideo Matsumoto

**Affiliations:** ^1^ Pharmacology Laboratories Taisho Pharmaceutical Co., Ltd. Saitama Japan; ^2^ Pharmaceutical Business Strategic Planning Taisho Pharmaceutical Co., Ltd. Tokyo Japan; ^3^ Institute for Integrated Sports Medicine Keio University School of Medicine Tokyo Japan

**Keywords:** arthritis, gait, NSAID patch, S (+)‐flurbiprofen plaster

## Abstract

**Objectives:**

We developed S (+)‐flurbiprofen plaster (SFPP), a novel NSAID patch containing S (+)‐flurbiprofen (SFP), a potent cyclooxygenase (COX) inhibitor. The purpose of this study was to assess efficacy of SFPP by analysing its effect on the gait disturbance and measuring the prostaglandin E_2_ (PGE_2_) production in synovial fluid in a rat model of knee arthritis.

**Methods:**

Knee inflammation was induced in rats by intra‐articular injection of a yeast suspension. Subsequently, an NSAID patch containing SFP, ketoprofen or loxoprofen was applied over the affected knee. Gait was assessed at 2, 4 and 6 h after application of the patch. The PGE_2_ concentration in the synovial fluid was measured after the gait assessment.

**Key findings:**

Application of SFPP (0.125, 0.25, 0.5 or 1 mg/sheet) was followed by a decrease in the visual gait score at all the doses examined. In the case of the other two NSAID patches, only the ketoprofen patch (1 or 2 mg/sheet) and loxoprofen patch (5 mg/sheet) produced a decrease in the visual gait score. All of the NSAID patches decreased the PGE_2_ production in the synovial fluid.

**Conclusions:**

These results suggest the potential usefulness of SFPP as an analgesic patch in patients with inflammatory joint pain.

## Introduction

Non‐steroidal anti‐inflammatory drugs (NSAIDs) are commonly used clinically for alleviation of inflammation and pain in patients with musculoskeletal disorders such as rheumatoid arthritis (RA) and osteoarthritis (OA). NSAIDs inhibit cyclooxygenase (COX), and their pharmacological and toxicological effects are attributed to their inhibiting the production of prostaglandins (PGs), including PGE_2_, a major mediator of inflammatory pain.^1^, ^2^ Topical application of NSAIDs has several advantages over oral administration of these agents, such as a lower risk of gastrointestinal adverse effects, protection of the active compound from gastric enzymes and avoidance of hepatic first‐pass metabolism.^3^ In clinical situations, however, skin absorption and penetration of active ingredients remain problematic issues in topical application of NSAIDs; hence, topical application often needs to be combined with oral administration of these drugs. Therefore, an NSAID patch that would allow sufficient absorption of the active ingredient and exert potent analgesic and anti‐inflammatory actions has been sought for the control of arthritis.

We developed a novel NSAID patch, S (+)‐flurbiprofen plaster (SFPP), containing S (+)‐flurbiprofen (SFP) as the active ingredient. Flurbiprofen (FP) in the form of a racemic mixture that has been widely used in therapeutics as an anti‐inflammatory and analgesic agent. SFP exerts a stronger inhibitory effect on COX than R (−)‐flurbiprofen (RFP), and the pharmacological effect of FP is predominantly attributable to SFP.^4^, ^5^ In addition, both animal and clinical studies have demonstrated a relatively higher skin permeability of FP as compared to that of other NSAIDs.^6^, ^7^ We previously demonstrated the superior skin permeability of SFP as compared to that of FP in the yucatan micropig (YMP) model *in vitro*.^8^ Another clinical study demonstrated superior percutaneous absorption and greater tissue penetration of the active ingredient of SFPP as compared to that of an FP patch.^9^ We demonstrated in a previous study that topical application of SFPP produced an immediate and strong analgesic effect in a rat model of adjuvant‐induced arthritis (AIA).^10^ Therefore, in view of its potent inhibitory effect on COX and good skin absorption, SFPP may be expected to exert a potent pharmacological effect.

The superior analgesic effect of SFPP as compared to that of the ketoprofen patch or loxoprofen patch has been demonstrated in a rat model of AIA.^10^ In clinical situations, the pain severity in patients with arthritis of the knee is usually evaluated by assessment of the spontaneous pain using a pain score, such as the Visual Analogue Scale (VAS). In contrast, in the AIA model, the analgesic effect is evaluated by measuring the amount of pain induced when the affected joint is passively moved. Thus, there is a discrepancy in the pain assessment method between clinical and non‐clinical situations. This discrepancy may result in the difficulty to predict effect of analgesics in an animal model. Hence, we need a new approach to assess pain in non‐clinical situations. Gait analysis can also be effectively used to assess movement‐evoked spontaneous pain in rat models of knee arthritis. The advantages of gait analysis in rat models are that the severity of pain can be evaluated in free‐moving animals, without application of a noxious stimulus. Although the quadrupedal gait patterns in rodents are clearly different from the bipedal patterns in humans, gait disturbance can be quantitatively analysed in a model of monoarthritis.^11^, ^12^

Evaluation of pain by gait analysis has been proposed to be mimic to evaluation of movement‐evoked pain in human patients with arthritis of the knee. Therefore, a drug showed analgesic effect in this evaluation can be expected to be effective knee pain associated with walking in clinical situation. However, ‘the analgesic efficacy of SFPP has not yet been examined in such an evaluation with a rat model of knee arthritis.’

Therefore, we aimed to evaluate the analgesic efficacy of SFPP using gait analysis. In the present study, we first established a method for analysis of gait as an assessment method for movement‐evoked pain in a rat model of yeast‐induced knee arthritis, which induced knee inflammation associated with PGE_2_. Then, we compared the analgesic efficacy of SFPP with that of other clinically available NSAID patches using this animal model. We also evaluated the target engagement of SFPP and other clinically available NSAID patches by measuring the inhibitory effect of the respective NSAIDs on the production of PGE_2_, in which COX plays an important role, in the synovial fluid.

## Materials and Methods

### Drugs and reagents

The following products were evaluated in this study: SFPP (40 mg/140 cm^2^, Loqoa^®^ tape; Taisho Toyama Pharmaceutical Co., Ltd., Tokyo, Japan), ketoprofen patch (40 mg/140 cm^2^; Hisamitsu Pharmaceutical Co., Inc., Tosu, Japan) and loxoprofen patch (100 mg/140 cm^2^; Daiichi‐Sankyo Co., Ltd., Tokyo, Japan). All the patches were purchased as commercially available products. Clinically, all of the drugs in this study were applied over the affected area in the form of a skin patch (10.0 × 14.0 cm). Brewer's yeast (Mitsubishi Tanabe Pharma Corporation, Osaka, Japan), indomethacin (Sigma‐Aldrich, St. Louis, MO, USA) and the PGE_2_ enzyme immunoassay (EIA) kit (Arbor Assays, Ann Arbor, MI, USA) were purchased from the companies indicated in the parentheses.

### Animals

Six‐week‐old male Sprague‐Dawley rats (Charles River Japan, Yokohama, Japan) were used for the gait analysis. The animals were housed under controlled temperature (23 ± 3°C), humidity (55 ± 20%) and lighting (lights on from 0700 to 1900 h) conditions. All of the animal experiments reported here were reviewed and approved by the Institutional Animal Care and Use Committee of Taisho Pharmaceutical Co., Ltd., and were in accordance with the Guidelines for Proper Conduct of Animal Experiments (Science Council of Japan, 2006).

### Measurement of gait

All the rats were placed on an acrylic wheel (11 cm in width and 40.8 cm in diameter) that was revolving at 3.5–4 rpm and trained to keep walking on it for 2 min a day, 3 times a week prior to the experiments. On the day prior the experiment, each rat was made to walk for 1 min on the revolving wheel. On the day of the experiment, each rat was anesthetized with isoflurane (Mylan, Canonsburg, PA, USA), followed by intra‐articular injection of a yeast suspension. The walking behaviour was video‐recorded from the bottom of the wheel with a high‐speed camera (GT‐03‐01; Noveltec Inc., Kobe, Japan). Gait was evaluated visually and graded semi‐quantitatively on a scale of 0–3, as shown in Table 1, as a consensus score between two experienced examiners. The evaluation was conducted in a blinded manner and always carried out by the same experimenter, to minimize variability. The mean value of the two data‐sets collected by the two experimenters was used as the visual gait score for each rat.

**Table 1:** Visual gait scores description/criteria

0	Normal
1	Slight difference in timing between right and left foot‐strike divided by the stride time
2	Limping, using toes only for steps, different time between a right and left foot‐strike divided by stride time
3	Dragging and carrying the leg, marked different time between a right and left foot‐strike divided by stride time

### Induction of knee arthritis and validation study

To determine the most suitable concentration of Brewer's yeast for producing the arthritis model for this study, rats were anesthetized and shaved the right knee, and then given a single intra‐articular injection of 5%, 10% or 20% Brewer's yeast suspension. Brewer's yeast was suspended in physiological saline and injected into the right knee at a volume of 30 μl using a 30‐gauge needle. Indomethacin (0.1, 1 or 10 mg/kg) was suspended in 0.5% methyl cellulose and administered orally prior to the intra‐articular injection of 10% yeast suspension. The group of rats that received intra‐articular injection of the yeast suspension was used as the control group. The group of rats that received intra‐articular saline injection was used as the saline group. Gait change was assessed 5 h after injection of the yeast suspension, and the synovial fluid PGE_2_ concentration was measured after the gait analysis.

### Measurement of the PGE_2_ level in the synovial fluid

The rats were anesthetized and sacrificed after the gait evaluation. The affected joint cavity was opened by cutting the upper part of the patella, and the exudate fluid was collected. In addition, joint lavage fluid was collected by instilling 13 μl of saline twice. The collected fluid specimen was centrifuged at 358*g* for 5 min and the supernatant was stored at −80°C until the PGE_2_ assay. The PGE_2_ concentration was measured using a PGE_2_ EIA kit and expressed as of percentage of the mean PGE_2_ level in the control group, as follows:

$${\mathrm{PGE}}_{2}\;\mathrm{level}\;\left(\%\;\mathrm{of}\;\mathrm{control}\right)=A/B,$$

where A is the PGE_2_ concentration in the synovial fluid in each treated group, and B is the average PGE_2_ concentration in the synovial fluid in the control group.

### Assessment of gait change after application of the drug patch

Inflammation of the rat right knee was induced by intra‐articular injection of a 10% yeast suspension. Each of the test patches was applied over the same knee immediately after the injection and covered with adhesive bandage tape to prevent the patch from falling off. The drug doses in all the patches were adjusted considering the clinically used doses and the difference in size between humans and rats, as follows: The SFPP patches contained SFP at 0.125, 0.25, 0.5 or 1 mg/sheet (patch sizes 0.5 cm × 0.875 cm, 1 cm × 0.875 cm, 1 cm × 1.75 cm and 2 cm × 1.75 cm, respectively); ketoprofen patches contained ketoprofen at 0.25, 0.5, 1 or 2 mg/sheet (patch sizes 1 cm × 0.875 cm, 1 cm × 1.75 cm, 2 cm × 1.75 cm and 2 cm × 3.5 cm, respectively); the loxoprofen patches contained loxoprofen at 0.625, 1.25, 2.5 and 5 mg/sheet (patch sizes 1 cm×0.875 cm, 1 cm × 1.75 cm, 2 cm × 1.75 cm and 2 cm × 3.5 cm, respectively). The control and saline groups received only application of an adhesive bandage not containing any active drug ingredient. Gait assessment was conducted at 2, 4 and 6 h post‐patch application. The area under the curve (AUC) of the pharmacological data was calculated by the area method from the visual gait score over a 0–6 h period.

### Data analysis

The data are expressed as means ± SE and all the statistical analyses were performed using the SAS software (SAS Institute Japan, Tokyo, Japan). The differences in the visual gait score between the saline group and control groups were tested for statistical significance using Wilcoxon test, and the differences between the control group and patch experimental groups were tested by Steel test. The differences in the AUC and synovial fluid PGE_2_ level between the saline group and control groups were tested for statistical significance using Student's *t*‐test or Welch's test after the *F*‐test, and the differences between the control group and patch experimental groups were tested by Dunnett's test. The significance levels were 1% (one‐sided) in the *F* test and 5% (two‐sided) in the other tests.

## Results

### Gait disturbance in a rat model of yeast‐induced knee arthritis

**Figure 1:**
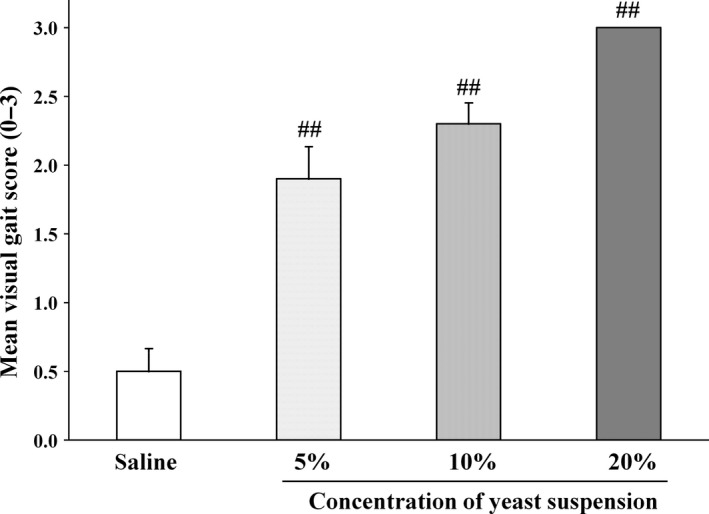
Intra‐articular injection of yeast suspension: the concentration–effect relationship. Rats were injected with 5%, 10% or 20% yeast suspension into the right knee. Change of visual gait score was assessed at 5 h after the injection of the yeast suspension. Each value and bar represent the mean ± SE of the results obtained in 10 animals. ^##^ *P* < 0.01 vs saline group (Steel test).

Intra‐articular injection of the yeast suspension resulted in a concentration‐dependent increase in the visual gait score (Figure 1). In all the yeast‐injected rats, the visual gait score was significantly increased at 5 h after the yeast injection as compared to the saline group. The average visual gait scores assessed after intra‐articular injection of 5, 10 and 20% yeast suspension were 1.9 ± 0.2, 2.3 ± 0.2 and 3.0 ± 0.0, respectively. As these results revealed that the visual gait score reached its peak following injection of a 20% yeast suspension, 10% yeast suspension was selected as the most effective concentration for the validation study (See Video S1 shows the gait in the saline group and Video S2 shows the gait in the 10% yeast suspension group).

### Effects of indomethacin on the gait disturbance and synovial fluid PGE_2_ levels in the rat model of arthritis

**Figure 2:**
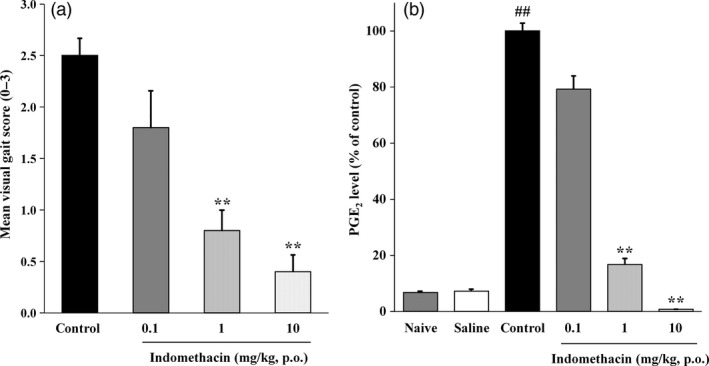
Effect of indomethacin on the gait disturbance induced by intra‐articular injection of a 10% yeast suspension (a) and on the synovial fluid PGE_2_ level (b). (a) Each value and bar represent the mean ± SE of the results obtained in 10 animals. **P < 0.01 vs control group (Steel test). (b) Rats were sacrificed, and the right hind paw was removed. The PGE_2_ concentration in the synovial fluid was measured by an EIA. Each value and bar represent the mean ± SE of the results obtained in 6 animals. ^##^ *P* < 0.01 vs saline group (Welch's t‐test). **P < 0.01 vs control group (Dunnett's test).

Oral administration of indomethacin significantly decreased the visual gait score in a dose‐dependent manner, and a significant difference in the score was observed at both the dose of 1 mg/kg and the dose of 10 mg/kg (Figure 2a). The average synovial fluid PGE_2_ concentration in the control group was 28.2 ± 2.7 ng/ml. Oral indomethacin also significantly decreased the synovial fluid PGE_2_ levels in a dose‐dependent manner, and a significant difference observed between the dose of 1 mg/kg and 10 mg/kg (Figure 2b).

From these results, we determined that intra‐articular injection of a 10% yeast suspension was appropriate for assessment of the effect of NSAID patch application on the gait disturbance and the following experiments were conducted under this condition.

### Effects of SFPP, ketoprofen patch and loxoprofen patch application on the visual gait score

**Figure 3:**
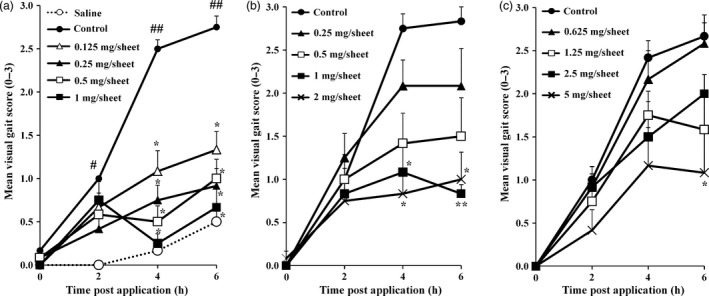
Time‐course of changes in the visual gait score after application of each patch (a) SFPP, (b) ketoprofen patch, (c) loxoprofen patch. A 10% yeast suspension was injected into the right knee and the gait changes were measured at 2, 4 and 6 h after the patch application. Each value and bar represent the mean ± SE of the results obtained in six animals. ^#^ *P* < 0.05, ^##^ *P* < 0.01 vs saline group (Wilcoxon's test). *P < 0.05, **P < 0.01 vs control group (Steel test).

**Figure 4:**
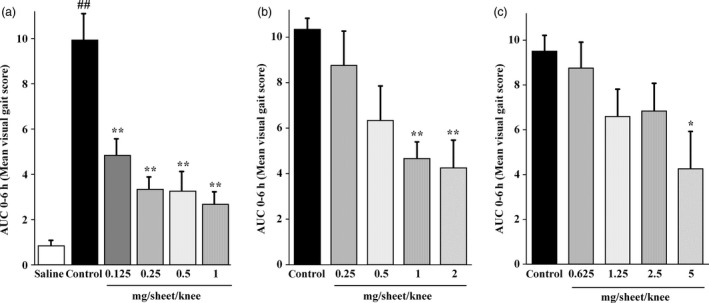
AUC of the change in the visual gait score from 0 to 6 h (a) SFPP, (b) ketoprofen patch (c) loxoprofen patch. Each value and bar represent the mean ± SE of the results obtained in six animals. ^##^ *P* < 0.01 vs saline group (Welch's t‐test). *P < 0.05, **P < 0.01 vs control group (Dunnett's test).

Figure 3 shows the changes of the visual gait scores from 0 to 6 h after application of SFPP, ketoprofen patch and loxoprofen patch. The visual gait score decreased significantly as compared to the score in the control group at 4 and 6 h after SFPP application at any dose (Figure 3a). It also decreased significantly at 4 and 6 h following application of the ketoprofen patch containing 1 or 2 mg of ketoprofen/sheet (Figure 3b), and at 6 h after application of loxoprofen patch containing 5 mg of loxoprofen/sheet (Figure 3c). The AUC is shown in Figure 4a–c. Thus, SFPP showed pharmacological effect at a lower dose of the active ingredient (0.125 mg of SFP/sheet) than the ketoprofen patch (1 mg ketoprofen/sheet) or loxoprofen patch (5 mg loxoprofen/sheet).

### Effects of SFPP, ketoprofen patch and loxoprofen patch application on the synovial fluid PGE_2_ levels

**Figure 5:**
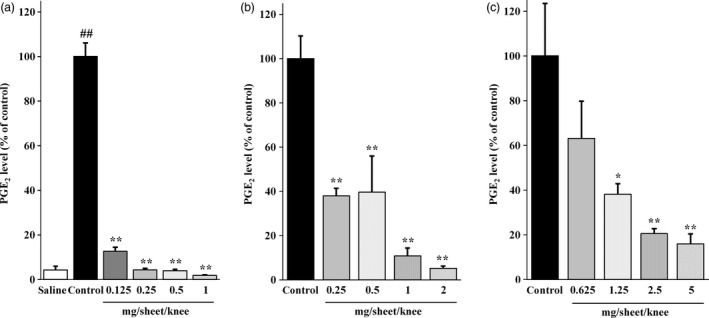
Effects of (a) SFPP, (b) ketoprofen patch and (c) loxoprofen patch application on the synovial fluid PGE_2_ level in the affected joint in the rat model of yeast‐induced inflammatory arthritis. Each value and bar represent the mean ± SE of the results obtained in six animals. ^##^ *P* < 0.01 vs saline group (Welch's t‐test). *P < 0.05, **P < 0.01 vs control group (Dunnett's test).

Figure 5a–c shows the inhibitory effects of SFPP, ketoprofen patch and loxoprofen patch application on the synovial fluid PGE_2_ levels. SFPP at any dose from 0.125 to 1 mg/sheet decreased the synovial fluid PGE_2_ levels. Application of SFPP containing 0.25–1 mg/sheet of SFP reduced the synovial fluid PGE_2_ levels to the levels recorded in the saline group. Decrease in the synovial fluid PGE_2_ levels was also observed following application of the ketoprofen patch (containing 0.25–2 mg/sheet of ketoprofen) and loxoprofen patch (containing 1.25–5 mg/sheet of loxoprofen). All the NSAIDs patches were ranked as follows in terms of the potency of their inhibitory effect on PGE_2_ production: SFPP > ketoprofen patch > loxoprofen patch.

## Discussion

The main purpose of this study was to compare the analgesic efficacy of SFPP with that of other clinically available NSAID patches. However, the conventionally used method for pain evaluation in animals is not very suitable for the evaluation of movement‐evoked spontaneous pain. Therefore, we first established a gait analysis method for the assessment of movement‐evoked spontaneous pain. We also evaluated the inhibitory effect of SFPP and other clinically available NSAID patches on the production of PGE_2_ in the synovial fluid. These results demonstrated the superior efficacy of SFPP as compared to other clinically available NSAID patches.

The rat model of yeast‐induced acute inflammatory pain is frequently used for evaluation of the analgesic efficacy of NSAIDs.^13^ Therefore, we considered that this model is the most suitable for assessing the analgesic efficacy of our novel NSAID patch and comparing it with the analgesic efficacies of other commercially available NSAID patches. First, we showed that the visual gait score in the rat model of yeast‐induced arthritis increased in a concentration‐dependent manner and that this increase in the score was suppressed by oral indomethacin administration. NSAIDs exert their pharmacological effects mainly through decreasing PGE_2_ production, a process in which COX plays an important role. PGE_2_ is a major mediator of inflammatory symptoms; in particular, it plays a pivotal role in the induction of peripheral hyperalgesia and allodynia.^2^ Knee inflammation induced by intra‐articular injection of a yeast suspension increased the synovial fluid PGE_2_ levels, which may result in knee hyperalgesia. Indomethacin also decreased the synovial fluid PGE_2_ levels at the same dose at which it improved the gait disturbance. Indomethacin is used as a classically effective NSAID and is reported to be effective at alleviating pain in various animal models of inflammatory pain.^14^, ^15^ We concluded that gait analysis is a valuable tool for the assessment of movement‐evoked pain in the rat model of yeast‐induced knee arthritis, that it is useful for assessing the analgesic efficacy of NSAIDs against arthritic pain.

Comparison of the effects of application of NSAID patches (SFPP, ketoprofen patch and loxoprofen patch) on the visual gait score revealed that SFP, the active ingredient in SFPP, exerted a pharmacological effect at a lower dose (0.125 mg/sheet) than ketoprofen (1 mg/sheet) or loxoprofen (5 mg/sheet) applied in patch form. In addition, comparison of the effects of application of the patches on the synovial fluid PGE_2_ levels showed that SFPP application produced a greater degree of decrease in the synovial fluid PGE_2_ levels than the ketoprofen patch or loxoprofen patch. Thus, SFPP was superior to the ketoprofen patch and loxoprofen patch, both in terms of its effect on the gait disturbance and in terms of its effect on the synovial fluid PGE_2_ levels. In clinical settings, SFPP is applied at a patch size of 10.0 cm × 14.0 cm over the affected area. Application of a 0.875‐cm^2^ SFPP in the rat model in this study was based on the difference in the body weight between humans and rats. SFPP showed the most superior pharmacological effects among the patches examined in this study (See Video S3–S5. Gait change in the rat model of yeast‐induced knee arthritis 6 h after in 0.875‐cm^2^ size of SFPP (Video S3), ketoprofen patch (Video S4) and loxoprofen patch (Video S5) application.) We have reported previously that application of SFPP provides relief to knee OA patients by alleviating the pain associated with walking and ascending or descending stairs.^16^ Among the three patches of 0.875‐cm^2^ size used in this study, SFPP was found to be the most effective in alleviating the gait disturbance in the rat model of yeast‐induced knee arthritis. Since gait disturbance in the rat model of knee arthritis has been proposed to mimic the clinically observed symptom of joint pain associated with walking in patients with arthritis of the knee, SFPP can be expected to be the most effective NSAID patch for knee pain associated with walking in clinical settings.

The reasons underlying the superior efficacy of SFPP in improving gait disturbance and decreasing the synovial fluid PGE_2_ levels in the rat model of yeast‐induced knee arthritis are summarized as follows: (1) SFP potently inhibits COX‐1 and COX‐2; (2) skin permeability of the active ingredient in SFPP is greater than that of the NSAID drugs in other NSAID patches. In a previous study, we reported that SFP inhibited human COX‐1 and COX‐2 more potently than ketoprofen and loxoprofen.^10^ Some reports have indicated that the S‐enantiomers of arylpropionic NSAIDs are the pharmacologically active enantiomers. Differences in the pharmacological effects and skin permeability are observed between racemates and enantiomers.^17^, ^18^ We also showed that SFP was a 1000‐fold more potent than RFP at inhibiting PGE_2_ production from peritoneal leukocytes stimulated with bacterial suspensions (IC_50_ = 14 nm for SFP and 17 000 nm for RFP, respectively^8^) Furthermore, we demonstrated that the skin permeability of SFP, the active ingredient of SFPP, was superior to that of ketoprofen or loxoprofen contained in the respective patches in Healthy SD rats, and also that application of SFPP was associated with the most potent analgesic efficacy and inhibitory effect on PGE_2_ production in the AIA model among the three patches compared.^10^ Based on a clinical study, we reported that in knee OA patients, a higher drug penetration into the synovial tissues was observed for SFPP as compared to the FP patch.^9^ PGE_2_ production is increased in the synovial fluid of OA patients.^19^ We considered that SFPP improved the gait disturbance in the knee arthritis model because of the greater skin permeability of SFP and stronger inhibitory effect of SFP on PGE_2_ production as compared to the active ingredients in the other NSAID patches used in this study.

## Conclusion

We demonstrated in this study that SFPP significantly improved the gait disturbance in a rat model of yeast‐induced knee arthritis and that it was superior to the ketoprofen patch and loxoprofen patch. This was considered to be attributable to the potent inhibitory effect of SFP on the release of pain mediators and the good skin permeability of the active ingredient in SFPP. We showed that SFPP improved that gait disturbance in the yeast‐induced knee arthritis model at the dose revealed clinical dose of NSAIDs patch in human and that SFPP exerted superior pharmacological effects as compared to other NSAID patches. Pain during walking in arthritis patients, especially patients with OA, is thought to contribute considerably to deterioration in the quality of life (QOL), and SFPP could be useful for the treatment of arthritic pain in clinical settings.

## Declarations

### Conflict of interest

A. Fukumoto, K. Tajima, M. Hori, Y. Toda, S. Kaku are employees of Taisho Pharmaceutical Co., Ltd. and H. Matsumoto receives consulting fees from Taisho Pharmaceutical Co., Ltd.

## Supporting information


**Video S1**. Gait in the saline group.

**Video S2**. Gait in the rat model of yeast‐induced knee arthritis.

**Video S3**. Gait in the rat model of yeast‐induced knee arthritis 6 h after in 0.875‐cm^2^ size of SFPP application.

**Video S4**. Gait in the rat model of yeast‐induced knee arthritis 6 h after in 0.875‐cm^2^ size of ketoprofen patch application.

**Video S5**. Gait in the rat model of yeast‐induced knee arthritis 6 h after in 0.875‐cm^2^ size of loxoprofen patch application.
